# Rheumatism and Gout

**Published:** 1890-09

**Authors:** 


Rheumatism and Gout. By F. LeRoy Satterlee, M. D., Ph. D., Professor of Chemistry, Materia Medica and Therapeutics in the New York College of Dentistry; late Professor of Chemistry and Hygiene American Veterinary College; Attending Physician St. Elizabeth Hospital, New York, etc., etc., etc. Physician's Leisure Library, Series iv. 1890. Pp. 83. George S. Davis, Detroit, Mich. Price, in paper, 25 cents; in cloth, 50 cents.

This is an agreeably written little book which, as announced in the preface, contains nothing new as regards etiology, pathology, or treatment of the subjects under discussion. It does, however, place before us in a forcible manner what we actually know in regard to gout, rheumatoid arthritis, and the various forms of rheumatism.

The author does not enter into any discussion in regard to the microbal origin of rheumatism, but simply states that the specific microbe has not yet been discovered, and that, for the present, we cannot go back of the presence of an excess of uric acid and fibrin in the blood as a cause of the clinical phenomena of this disease.

The treatment that naturally follows from this pathogenesis is an avoidance of the nitrogenous foods and the use of alkalies.

He objects to the use of the salicylates in acute rheumatism. He says:

> The effect of these drugs, though at times brilliant in producing subsidence of pain, is very uncertain and always transient, relapses being the rule; and the continued administration of these remedies is, moreover, productive of a series of deleterious symptoms, such as gastric and cerebral disturbances, cardiac depression, obliteration of the first sound of the heart, anemia, and other toxic phenomena.

Our experience has been quite the opposite in the use of these remedies. In all cases of acute rheumatism it is our practice to give sodium salicylate in twenty grain doses every three or four hours, according to the severity of the symptoms. If, at the end of forty-eight hours of this treatment, the patient is not markedly improved, the carbonate of potassium is given in an effervescing draught, with lemon juice. We have seldom found that a resort to the potassium carbonate has to be made. Moreover, with proper care in not allowing the patient to get up too soon, the cardiac complications and sequelæ are very much less in cases treated with the salicylates than in those treated otherwise.

The fact that, in addition to its specific effect upon the rheumatism itself, the sodium salicylate possesses marked antipyretic and analgesic properties, makes recourse to other drugs for the production of these beneficial effects unnecessary, and so lessens the chances for gastric disturbance. We have not yet found the stomach that could not retain sodium salicylate if dissolved in simple syrup and *strongly* flavored with wintergreen (methyl salicylate).

The author's chapters on chronic rheumatism command our thorough approval.

Those on rheumatoid arthritis are also good.

To us the use of colchicum in gout has always seemed thoroughly empyrical and without scientific basis, and we are glad to quote Dr. Satterlee's protest against the administration of colchicum in any of its forms:

> . . . . . . Its effects are transient; it interferes with the digestion and circulation, induces muscular feebleness, and acts as an insidious general depressant. Patients soon learn to use it themselves, flying to it while persisting in the error of living, which renders it necessary to their relief. The writer has no hesitation in stating a conclusion, evolved from observation, that many of the sudden deaths among the wealthier classes, of men between fifty and sixty, ascribed to that vague causation, heart failure, are due to the improper use of colchicum.

The book, on the whole, is well worth perusal, and contains several valuable hints in regard to treatment. DeL. R.

